# Downregulation of PDIA4 inhibits proliferation and migration in human oral squamous cell carcinoma

**DOI:** 10.1186/s41065-025-00594-2

**Published:** 2025-11-03

**Authors:** Yue Hu, Wei Zhang, Fuyu Zhang, Qiaoyun Liu, Hao Yang

**Affiliations:** 1 Department of Radiation Oncology, Peking University Cancer Hospital (Inner Mongolia Campus) & Affiliated Cancer Hospital of Inner Mongolia Medical University, No.42 Zhaowuda Road, Saihan District, Hohhot, Inner Mongolia Autonomous Region 010020 China; 2 Graduate School, Inner Mongolia Medical University, Hohhot, Inner Mongolia Autonomous Region 010110 China

**Keywords:** Oral squamous cell carcinoma, PDIA4, Bioinformatics analysis, Prognosis and oncogene

## Abstract

**Background:**

Protein disulfide isomerase family A member 4 (PDIA4), a member of the protein disulfide isomerase family, has been associated with the progression of cancer. Nevertheless, its specific function in oral squamous cell carcinoma (OSCC) is not yet well understood.

**Methods:**

To assess the prognostic significance and functional profile of the PDIA4, survival analysis and GSEA were conducted. Additionally, we examined the differences in immune infiltration and immunotherapy response between groups with low and high expression levels of PDIA4. Subsequently, RT-qPCR and western blot assays were employed to verify PDIA4 expression in OSCC tissues. The functional implications of PDIA4 in OSCC cells were also investigated.

**Results:**

Analysis of the TCGA-OSCC dataset revealed a notable increase in PDIA4 expression in OSCC tissues, as verified by RT-qPCR and western blot analyses. Additionally, elevated PDIA4 levels were associated with poor prognosis in OSCC patients. GSEA results showed that the cellular senescence, FoxO and Hippo signaling pathways were remarkably inactivated in the high PDIA4 expression group. Moreover, a negative correlation was observed between PDIA4 levels and the infiltration of CD4, CD8 and natural killer T cells. Conversely, a positive correlation was observed between PDIA4 levels and M0 macrophage and regulatory T cell infiltration. Meanwhile, OSCC patients exhibiting elevated PDIA4 expression demonstrated elevated TIDE scores, implying a reduced responsiveness to immunotherapy in these individuals. Functionally, the suppression of PDIA4 significantly suppressed both proliferation and migration of OSCC cells, potentially through activating the FoxO1/p21^CIP1^ pathway.

**Conclusion:**

These findings suggest that PDIA4 may potentially serve as both a prognostic biomarker and a therapeutic target for OSCC patients.

**Supplementary Information:**

The online version contains supplementary material available at 10.1186/s41065-025-00594-2.

## Introduction

Oral squamous cell carcinoma (OSCC) is generally regarded as one of the most common malignant epithelial tumors in head and neck cancer, comprising over 90% of all head and neck cancer [1, 2]. OSCC typically arises from the oral mucosa’s squamous epithelium [3, 4]. It is capable of affecting various areas of the mouth, such as the tongue, gums, buccal mucosa, floor of the mouth, palate, and lips [3, 4]. The primary risk factors associated with OSCC include chewing betel nut, smoking, drinking alcohol and infection with human papillomavirus [5, 6]. As highlighted by Sonkin and Thomas et al., the evolution of cancer treatments—from surgery, radiation and chemotherapy to targeted therapies and immunotherapy—has transformed outcomes for many malignancies [7]. However, due to its typically subtle symptoms, OSCC is often diagnosed at advanced clinical stage [8, 9]. The overall five-year survival rate for OSCC patients remains low [10]. Consequently, to improve the prognosis for OSCC patients, it is crucial to identify reliable biomarkers and therapeutic targets for OSCC.

Protein disulfide isomerases (PDIs) are a group of oxidoreductases that facilitate redox-dependent protein folding and ensure quality control within the endoplasmic reticulum [11, 12]. Research has indicated that members of the PDI family are abnormally expressed in certain cancer types and influence cancer progression [13–15]. For example, protein disulfide isomerase family A member 3 (PDIA3) expression is notably elevated across various tumor types, and is closely linked to the prognosis of most cancers, such as Glioblastoma multiforme (GBM) and uveal melanoma [15]. Meanwhile, PDIA3 is capable of facilitating the polarization of M2 tumor-associated macrophages through activating the STAT3/PD-1 signaling, thereby greatly enhancing the progression of colorectal cancer [13]. Ji et al. reported that PDIA5 may also act as an oncogene, augmenting the invasive potential of GBM cells [16]. PDIA4, a member of the PDI family, is implicated in the development of various diseases, such as cancer [17], diabetic kidney disease [18], and infectious diseases [19, 20]. Evidence has shown that PDIA4 has dual functions in tumors; it can exert anti-tumor effects while also potentially promoting tumor development [17, 21]. For instance, Kim et al. discovered that downregulation of PDIA4 led to elevated cell proliferation and migration capacity in lung cancer cells [21]. Conversely, wang et al. reported that PDIA4 overexpression could facilitate glioblastoma cell proliferation through the activation of the PI3K/AKT signaling [17]. Additionally, Moreover, elevated levels of PDIA4 were closely linked to worse overall survival in cervical cancer [22]. This evidence highlights that the role of PDIA4 in tumor might depend on the specific tumor type. However, the impact of PDIA4 on prognosis in patients with OSCC remains uncertain, and its biological function in OSCC is not yet well understood.

Recently, comprehensive bioinformatics techniques for analyzing public databases have been utilized for the identification of prognostic biomarkers [23, 24]. This approach significantly facilitates the exploration of critical biological data. Accordingly, this study examined the transcriptional levels, prognostic significance, biological roles, and the association with immune infiltration of the PDIA4 gene in OSCC patients using bioinformatics analysis. Additionally, we also explored the biological function of PDIA4 in OSCC cells. These results may offer a potential strategy for OSCC treatment.

## Materials and methods

### Data collection

The head and neck squamous cancer (HNSCC) dataset, referred to as the TCGA-HNSCC dataset, was acquired from The Cancer Genome Atlas (TCGA, https://tcga-data.nci.nih.gov/tcga/) database. In this study, a total of 361 samples were retrieved from the TCGA-HNSCC dataset, which included 329 samples diagnosed with OSCC and 32 normal samples. The 329 cancerous samples originated from the alveolar ridge, root of the tongue, buccal mucosa, floor of the mouth, hard palate, oral cavity, and the oral tongue of OSCC patients. This subset is specifically referred to as the TCGA-OSCC dataset. A total of 328 samples with full clinical information were included for survival analysis.

The datasets numbered GSE30784 and GSE65858 were obtained from the Gene Expression Omnibus (GEO database, https://www.ncbi.nlm.nih.gov/geo/) database. GSE30784 dataset consists of 212 samples, comprising 167 OSCC samples and 45 normal oral samples. GSE65858 dataset contains 270 HNSCC samples, all of which include complete survival information.

### Differential expression analysis

Genes exhibiting differential expression between two groups were identified using the “DESeq2” package in R language [25]. Differentially expressed genes (DEGs) were determined based on the following criteria: |Log2FC| >1 and FDR < 0.05.

### Gene Ontology (GO), Kyoto Encyclopedia of Genes and Genomes (KEGG), Gene Set Enrichment Analysis (GSEA), and Gene Set Variation Analysis (GSVA)

The “clusterProfiler” package (version 4.8.3) in R language was employed for the enrichment analysis of GO (including Biological Process (BP), Molecular Function (MF) and Cellular Component (CC)) and KEGG pathway [26]. A *P*-value of < 0.05 was applied to identify significantly enriched GO terms and KEGG pathways.

GSEA analysis was conducted between two groups utilizing the “clusterProfiler” package (version 4.8.3) in R language [26]. Pathways were assessed for significant enrichment with a p.adjust threshold of less than 0.05.

GSVA analysis was conducted employing the “GSVA” package in R language for calculating the enrichment scores [27]. Next, analysis of differences between groups were conducted using the limma (version 3.56.2) package in R language [28]. The gene set of “c2.all.v2024.1.Hs.symbols” from the Molecular Signature Database was utilized as the reference set. Pathways meeting a *p*.adjust < 0.05 were regarded as significantly enriched.

### Univariate/multivariate COX regression analysis

Univariate Cox regression analysis was performed on PDIA4, utilizing a threshold of *P* < 0.05 to identify whether PDIA4 is linked to the prognosis of OSCC. Multivariate Cox regression model was applied to determine whether PDIA4 could independently predict the survival of OSCC patients, independently of other variables.

### Survival analysis

Survival rates between groups were assessed employing the “survival” and “survminer” packages in R, utilizing the Kaplan-Meier method. The significance of the difference in survival rates between groups was tested using the log-rank test.

### Immune infiltration analysis

The CIBERSORT software was used to determine the relative proportions of 22 immune cell types in the samples [29]. Meanwhile, the ESTIMATE package in R language was conducted to predict the levels of stromal and immune cell infiltration in tumor tissues [30].

### Drug sensitivity analysis

The “oncopredict” package in R language was utilized to conduct drug sensitivity prediction [31]. Using the GDSC2_expression matrix (including 17419 genes and 805 cell lines) and the GDSC2_drug sensitivity data (including IC50 values of 198 drugs in 805 cell lines) as the training sets, the IC50 values of the samples to be predicted were calculated. This was achieved using the “calcPhenotype” function based on the expression matrix of the samples.

### Clinical samples

Fifteen pairs of OSCC tissues from OSCC patients, along with their matched normal tissues, were collected from the Key Laboratory of Industrial Fermentation Microbiology of the Ministry of Education, College of Biotechnology, Tianjin University of Science and Technology. The study received approval from the Ethical Committee of Tianjin University of Science and Technology (grant no. YKD202301119) in accordance with the Declaration of Helsinki, and informed consent was obtained from all participants.

### RT-qPCR

Total RNA was extracted using the TriQuick Reagent and subsequently reverse-transcribed to create cDNA utilizing the SureScript™ First-Strand cDNA Synthesis Kit (GeneCopeia). This cDNA was then utilized as a template for the quantitative PCR process. The quantitative PCR was conducted using the 2×SYBR Green qPCR Master Mix kit (Servicebio). The results obtained were evaluated using the 2^−ΔΔCT^ method [32]. The primers used in this study was listed in Table 1.

**Table 1:** Primer sequences for qPCR

Genes	Forward Primer (5’−3’)	Reverse Primer (5’−3’)
PDIA4	GGCGAAGTAGATGGTGGG	AGGTCGTGGTGGGAAAGA
GAPDH	CAGGAGCGAGACCCCACTAA	ATCACGCCACAGCTTTCCAG

### Western blot

Proteins were isolated utilizing 10% SDS-PAGE gels, which were then electrostatically transferred to PVDF membranes. Following this, the membranes were allowed to incubate overnight at 4 °C with primary antibodies: anti-PDIA4 (ab190348, Abcam), anti-Cyclin E1 (CCNE1, 11554-1-AP, Proteintech), anti-CDK2 (10122-1-AP, Proteintech), anti-FOXO1 (18592-1-AP, Proteintech), anti-p21 (28248-1-AP, Proteintech) and anti-β-actin (ab8227, Abcam). Following completing the incubation with the appropriate secondary antibodies, the visualization of protein bands was successfully carried out by employing an ECL reagent.

### Cell culture and transfection

Human oral epithelial cells (HOECs) and OSCC cell lines HSC-3, TCA-8113 and CAL-27 were maintained in DMEM supplemented with 10% heat-inactivated FBS and 1% penicillin/streptomycin. All cells were cultured at 37 °C environment with 5% CO_2_.

For transient transfection, the siRNA negative control (si-NC) and PDIA4 siRNAs (si-PDIA4-1, si-PDIA4-2 and si-PDIA4-3) were transfected into CAL-27 cells using the Lipo2000 reagent.

### Cell counting kit-8 (CCK-8)

CAL-27 cells were placed in a 96-well plate at 37℃. Subsequently, 10 µL of CCK-8 solution (Beyotime) was added to every well, and cells were then incubated for 1 h. The absorbance for each well was recorded at 450 nm using a microplate reader.

### Colony formation assay

CAL-27 cells were placed in 12-well plates and incubated for a period of two weeks. When the number of cells per clone surpassed 50, each well was subjected to fixation with 4% paraformaldehyde and subsequently stained with 0.1% crystal violet. A camera was then used to capture the results.

### Wound healing assay

CAL-27 cells were placed in 6-well plates and incubated overnight. A scratch in the center of each well was carefully made using a sterilized pipet tip. Meanwhile, images were captured with a light microscope at 0, 24, or 48 h.

### Cellular senescence analysis

To assess cell senescence, a senescence-associated beta-galactosidase (SA-β-gal) staining kit from Solarbio was utilized. Following fixation, CAL-27 cells were incubated with the SA-β-gal working solution at 37 °C overnight. A microscope was used for capturing blue-stained senescent cells.

### Statistical analysis

For the bioinformatics data, the Wilcoxon rank sum test was employed to assess differences in gene expression and immune cell infiltration between groups. Pearson correlation analysis was performed using Pearson correlation coefficients. Differences were considered statistically significant at *p* < 0.05. All statistical analyses were performed using R software version 4.3.3.

For the experimental data, either Student’s t -test or one-way ANOVA was utilized to analyze differences among the groups. Data were expressed as mean ± SD, with *p* values < 0.05 considered statistically significant.

## Results

### PDIA4 is upregulated in OSCC tissues

**Fig. 1:**
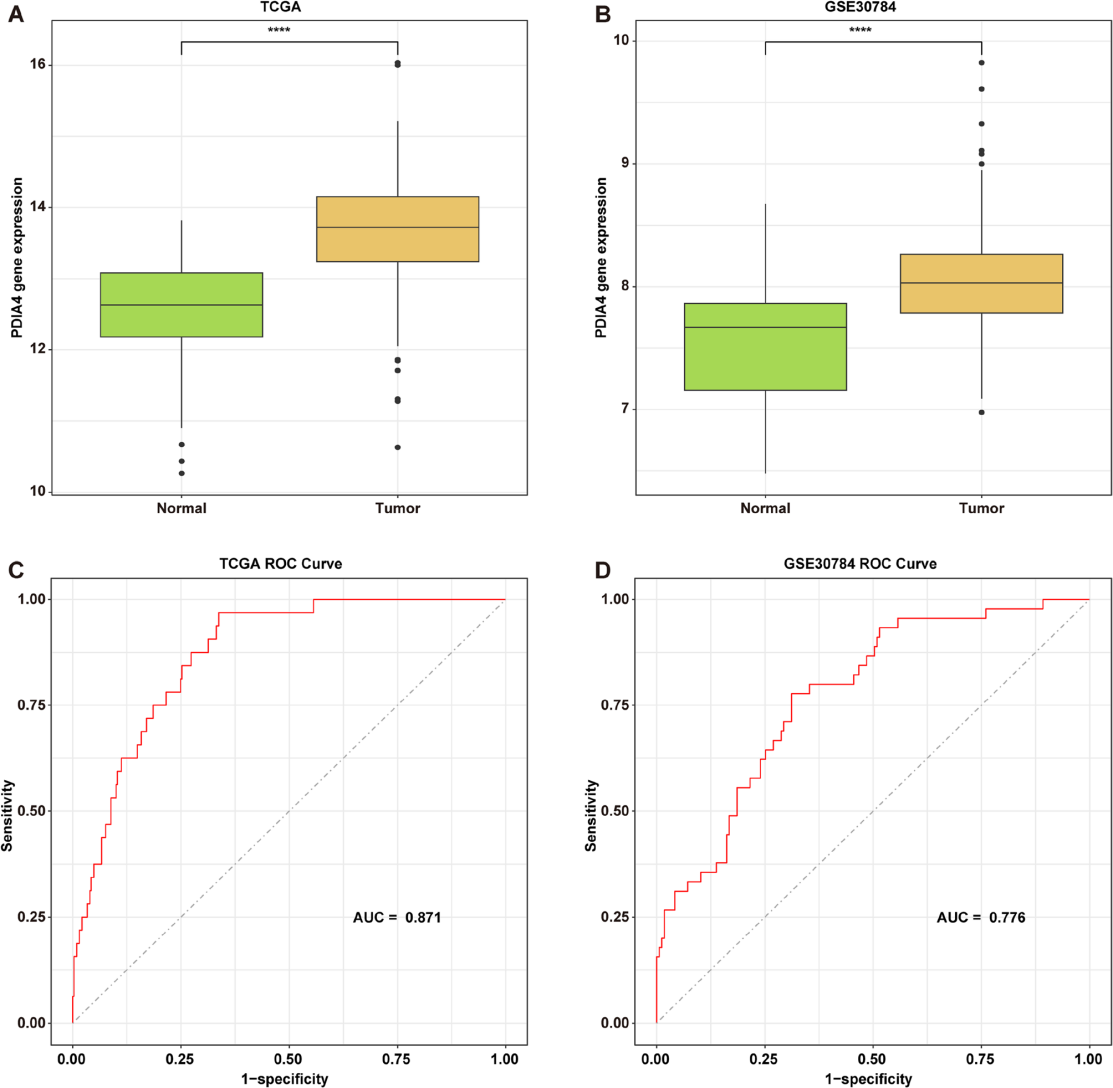
PDIA4 is upregulated in OSCC tissues. **A**,** B** Box plots of the comparison of PDIA4 expression between normal and tumor tissues in the **A** TCGA-OSCC and **B **GSE30784 datasets. ****P < 0.0001.** C**,** D **ROC diagnostic curve of PDIA4 in the **C **TCGA-OSCC and **D** GSE30784 datasets

Utilizing the data from the TCGA-OSCC and GSE30784 datasets, we observed that PDIA4 levels were notably higher in OSCC tissues compared to those in normal controls (Fig. 1A and B). Next, ROC curves were generated using the pROC package in R and area under the curve (AUC) values were computed to assess the possible diagnostic utility of PDIA4 in OSCC [33]. The AUC for the ROC curve of PDIA4 in the TCGA-OSCC cohort was 0.871, while the AUC for the GSE30784 dataset was 0.776 (Fig. 1C and D). These results showed that PDIA4 may serve as a potential diagnostic marker for OSCC.

### Elevated levels of PDIA4 associates with worse prognosis of OSCC patients

**Fig. 2:**
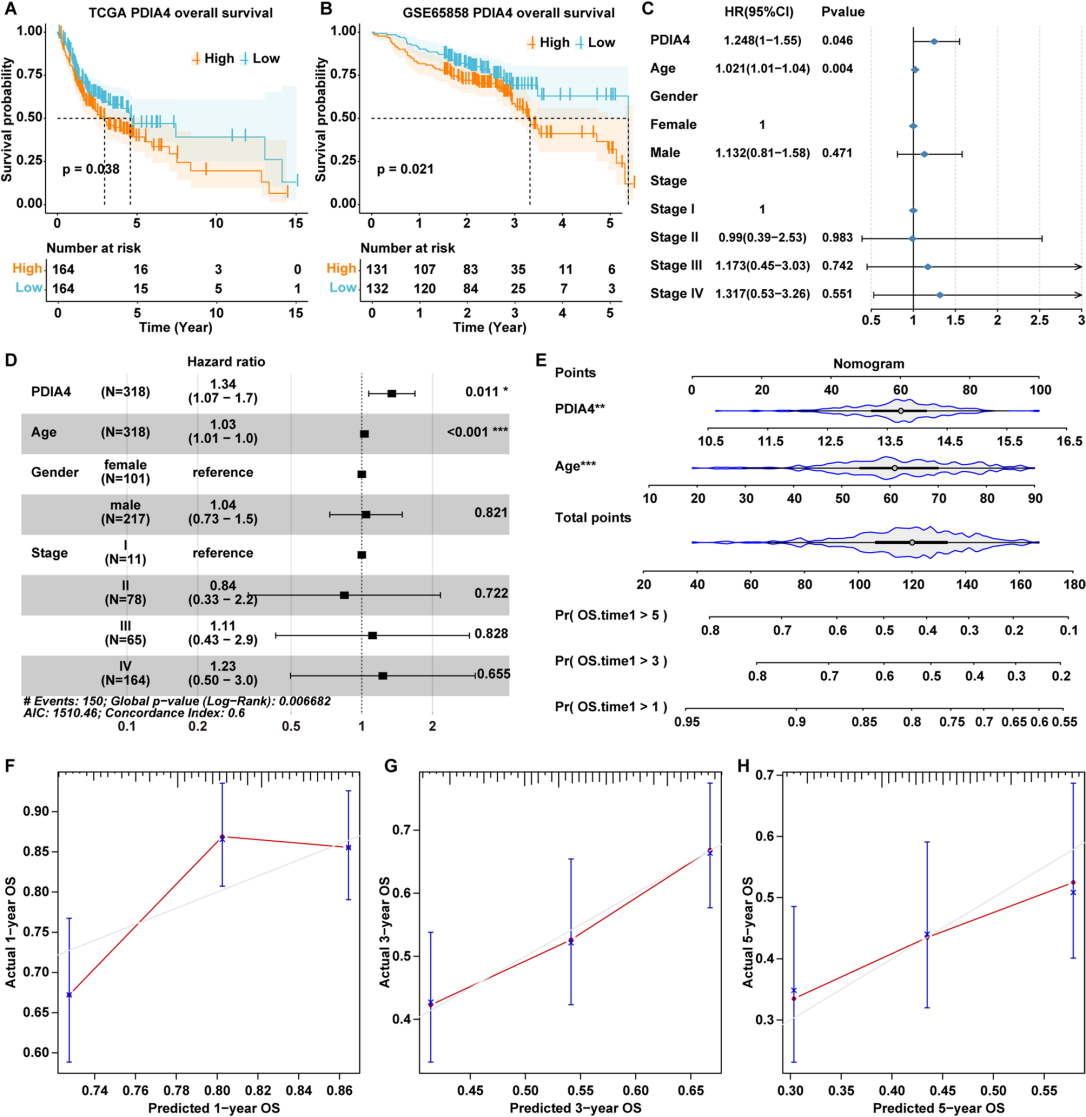
Elevated levels of PDIA4 associates with worse prognosis of OSCC patients. **A**, **B** K-M survival curves of OSCC patients in H-PDIA4 and L-PDIA4 groups in the **A** TCGA-OSCC and **B** GSE65858 datasets. **C** Univariate and **D** multivariate Cox regression analysis of PDIA4 expression for overall survival in the TCGA-OSCC dataset. *P < 0.05; ***P < 0.001. **E** Nomogram construction. **P < 0.01; ***P < 0.001. **F**, **G**, **H** Calibration curves of nomogram for 1, 3 and 5 years

Utilizing the TCGA-OSCC cohort, the samples were categorized into two groups based on the median expression level of PDIA4: PDIA4 high expression (H-PDIA4) group and PDIA4 low expression (L-PDIA4) group. Subsequently, a survival analysis was performed to compare the two groups. It was observed that OSCC patients in the H-PDIA4 group exhibited a lower survival rate (Fig. 2A). Similarly, the data from a HNSCC dataset GSE65858 also showed that the H-PDIA4 group also experienced a diminished survival probability (Fig. 2B).

We then conducted a univariate Cox regression analysis to examine the prognostic effects of several variables including age, gender, stage, and PDIA4 expression within the TCGA-OSCC cohort. The results showed that both PDIA4 expression and age were significant prognostic factors (Fig. 2C). Additionally, a multivariate Cox regression analysis utilizing data from the TCGA-OSCC cohort, which included PDIA4 expression along with age, gender and stage variables, demonstrated that PDIA4 expression functioned as an independent prognostic indicator (Fig. 2D). By integrating PDIA4 expression and age, we developed a Nomogram model to predict the 1-year, 3-year, and 5-year survival rates for OSCC patients. The results showed that a higher total score represented a reduced overall survival rates of OSCC patients (Fig. 2E). As shown in Fig. 2F-H, the 3-year and 5-year calibrated curves showed closer alignment with the ideal curve, indicating that the model’s predicted outcomes coincided well with the actual results.

To further investigate the prognostic significance of PDIA4, a subgroup analysis was performed using the TCGA-OSCC cohort, stratified by age, gender and stage. Kaplan-Meier analysis indicated that high PDIA4 expression was associated with a lower survival rate in the subgroup of patients aged ≥ 61 years and in the female subgroup (*P* < 0.05), whereas no statistically significant differences were found in any of the other subgroups (*P* > 0.05) (Fig. S1A-H).

### Functional enrichment analysis of PDIA4

**Fig. 3:**
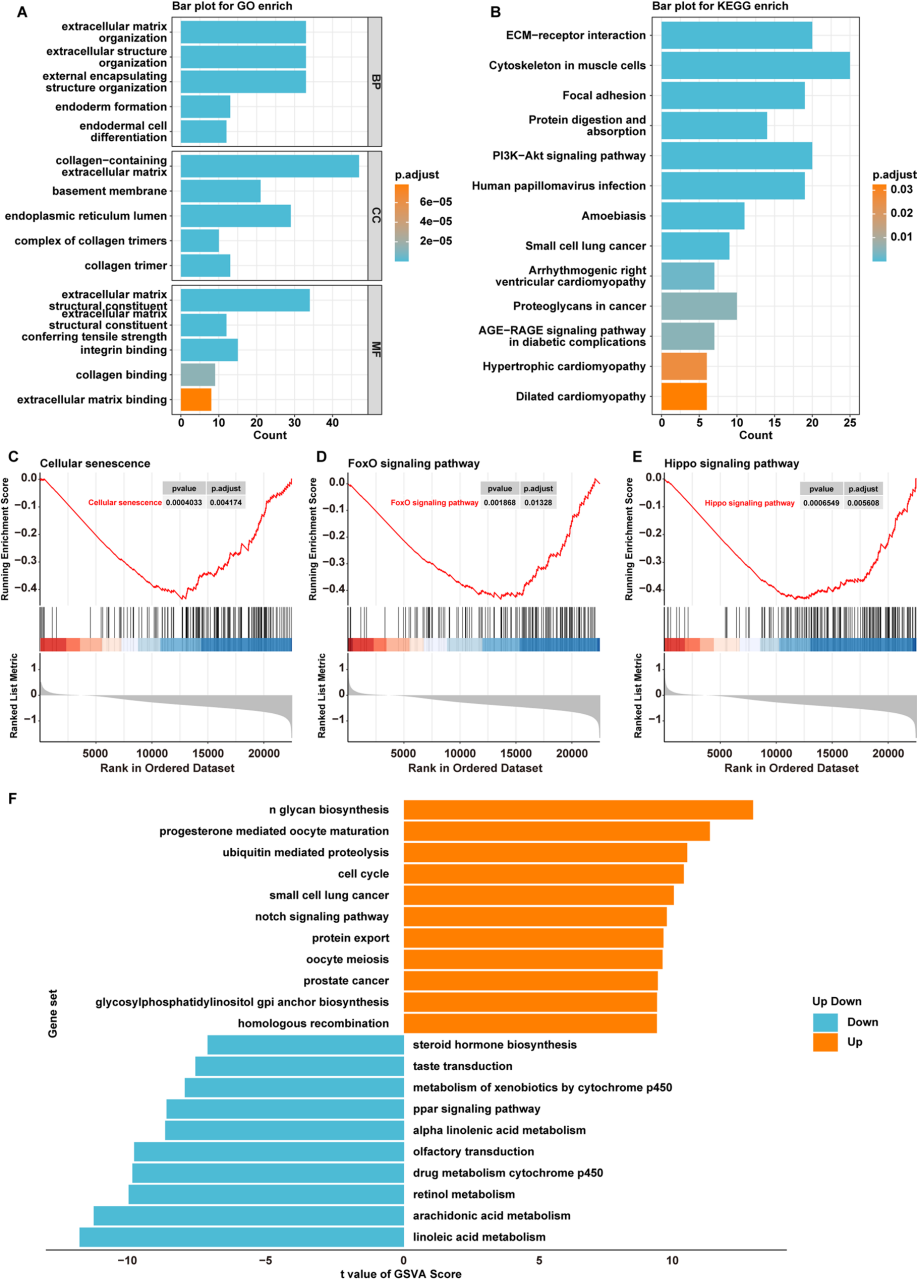
Functional enrichment analysis of PDIA4. **A** GO and **B** KEGG analyses of DEGs between H-PDIA4 and L-PDIA4 groups in the TCGA-OSCC dataset. **C**, **D**, **E** GSEA analysis of three pathways between H-PDIA4 and L-PDIA4 groups: **C** Cellular senescence, **D** FoxO signaling pathway and **E** Hippo signaling pathway. **F** The results of GSVA between H-PDIA4 and L-PDIA4 groups

Differential expression analysis was conducted to identify DEGs between H-PDIA4 and L-PDIA4 groups. Subsequently, GO and KEGG enrichment analyses were performed on these DEGs. A total of 30 KEGG pathways, 138 GO-BP terms, 21 GO-MF terms and 23 GO-CC terms were found to be significantly enriched (Fig. 3A and B, Table S1).

Meanwhile, GSEA enrichment analysis was performed between H-PDIA4 and L-PDIA4 groups, and the results showed that compared to L-PDIA4 groups, 76 pathways were remarkably enriched and inactivated in the H-PDIA4 group, including Cellular senescence, FoxO signaling pathway and Hippo signaling pathway (Fig. 3C-E and Table S2).

Furthermore, GSVA revealed significant differences in 134 KEGG pathways between H-PDIA4 and L-PDIA4 groups (Table S3). Among them, 96 pathways were significantly activated in the H-PDIA4 group, such as cell cycle and Notch signaling pathways, while 38 pathways were notably inactivated in the L-PDIA4 group, including various metabolic pathways (Fig. 3F and Table S3).

### PDIA4 is correlated with immune infiltration in OSCC

**Fig. 4:**
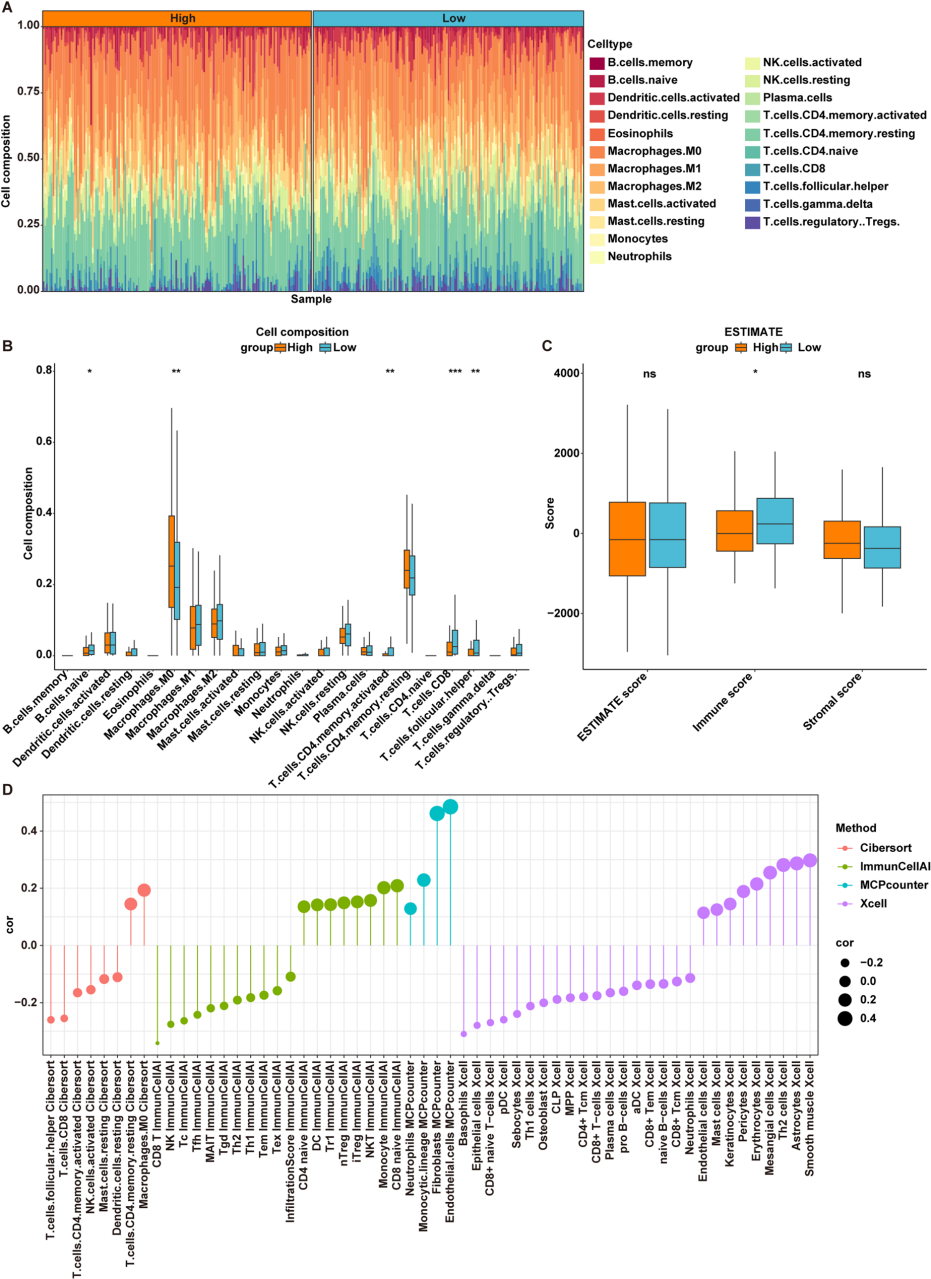
PDIA4 is correlated with immune infiltration in OSCC.** A** The 22 immune cell infiltration of OSCC samples assessed by CIBERSORT algorithm in the TCGA-OSCC dataset. **B** Box plots of the comparison of the expression levels of 22 immune cell types between H-PDIA4 and L-PDIA4 groups in the TCGA-OSCC dataset. *P < 0.05; **P < 0.01; ***P < 0.001. **C** Box plots of the comparison of the levels of ESTIMATE, immune and stromal scores between H-PDIA4 and L-PDIA4 groups in the TCGA-OSCC dataset. *P < 0.05. **D** Correlation analysis of immune cells and PDIA4 expression based on CIBERSORT, xcell, MCPcounter, ImmuneCellAI algorithms

The CIBERSORT algorithm was employed to calculate the relative abundance of 22 types of immune infiltrating cells present in OSCC samples from the TCGA-OSCC cohort (Fig. 4A). Furthermore, we analyzed the differences in immune cell infiltration among the 22 immune-infiltrating cell types between H-PDIA4 and L-PDIA4 groups. The results showed that, compared to the L-PDIA4 group, M0 Macrophages were notably increased, but naive B cells, activated memory CD4 T cells, CD8 T cells, and follicular helper T cells were markedly decreased in the H-PDIA4 group (Fig. 4B).

The levels of stromal/immune cell infiltration in tumor tissues were assessed using the ESTIMATE algorithm to compute stromal and immune scores. The results showed that there was no significant difference in stromal score and ESTIMATE score between the H-PDIA4 and L-PDIA4 groups. However, the immune score was significantly higher in the L-PDIA4 group compared to the H-PDIA4 group (Fig. 4C).

Subsequently, we employed CIBERSORT, Xcell, MCPcounter, ImmuneCellAI methods to further evaluate the proportion of immune cells between H-PDIA4 and L-PDIA4 groups. Overall, the infiltration of CD4 T cells, CD8 T cells and natural killer (NK) cells was notably negatively correlated with the H-PDIA4 group, while M0 Macrophages and Tregs showed a significant positive correlation with the H-PDIA4 group (Fig. 4D).

### PDIA4 is associated with immunotherapy efficacy in OSCC

**Fig. 5:**
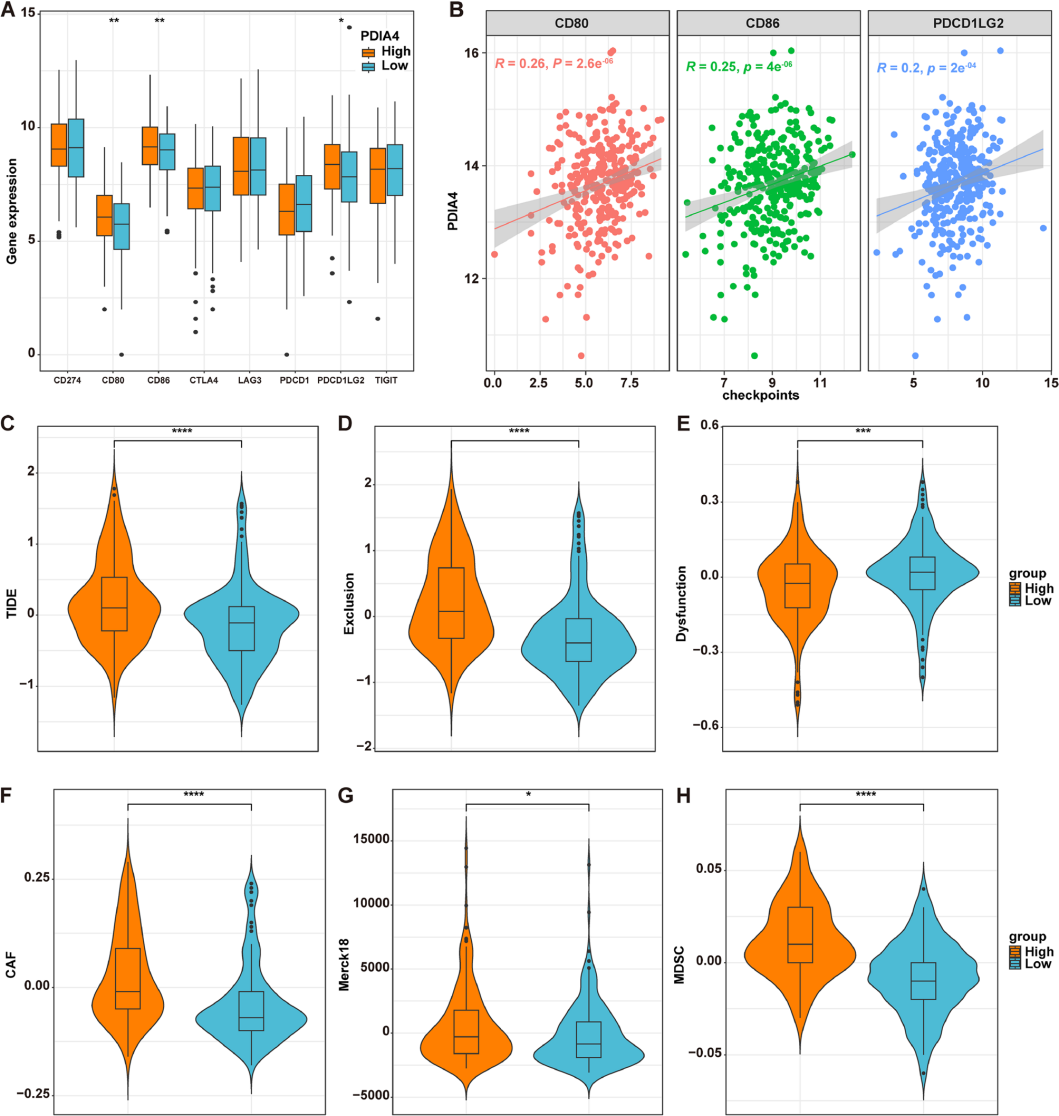
PDIA4 is associated with immunotherapy efficacy in OSCC. **A** Box plots of the comparison of the expression levels of eight immune checkpoints between H-PDIA4 and L-PDIA4 groups in the TCGA-OSCC dataset. *P < 0.05; **P < 0.01. **B** Correlation analysis of PDIA4 expression levels and CD80, CD86 and PDCD1LG2 expression levels. **C-H** Box plots of the comparison of the levels of **(C)** TIDE, **D** Exclusion, **E** Dysfunction, **F** CAF, **G** Merck18 and **H** MDSC scores between H-PDIA4 and L-PDIA4 groups in the TCGA-OSCC dataset. *P < 0.05; ***P < 0.001; ****P < 0.0001

It has been demonstrated that immune checkpoints (ICs) play key roles in mediating immune escape and hindering the effectiveness of cancer immunotherapy [34]. To assess the potential effects of PDIA4 on immunotherapy efficacy, we examined the levels of eight ICs between the H-PDIA4 and L-PDIA4 groups in the TCGA-OSCC cohort. The results indicated that the expression levels of CD80, CD86 and PDCD1LG2 were significantly higher in the H-PDIA4 group compared to the L-PDIA4 group (Fig. 5A). Meanwhile, PDIA4 expression exhibited a strong positive correlation with the levels of CD80, CD86 and PDCD1LG2, based on the data in the TCGA-OSCC cohort (Fig. 5B).

Furthermore, the TIDE algorithm employed to assess the response to immunotherapy. As illustrated in Fig. 5C-H, scores for TIDE, Exclusion, cancer-related fibroblasts (CAF), Merck18 and myeloid-derived suppressor cells (MDSC) were substantially elevated, while the Dysfunction score was obviously reduced in the H-PDIA4 group. These findings suggest that OSCC patients in the H-PDIA4 group may demonstrate reduced responsiveness to immunotherapy.

### The correlation between PDIA4 and drug sensitivity

**Fig. 6:**
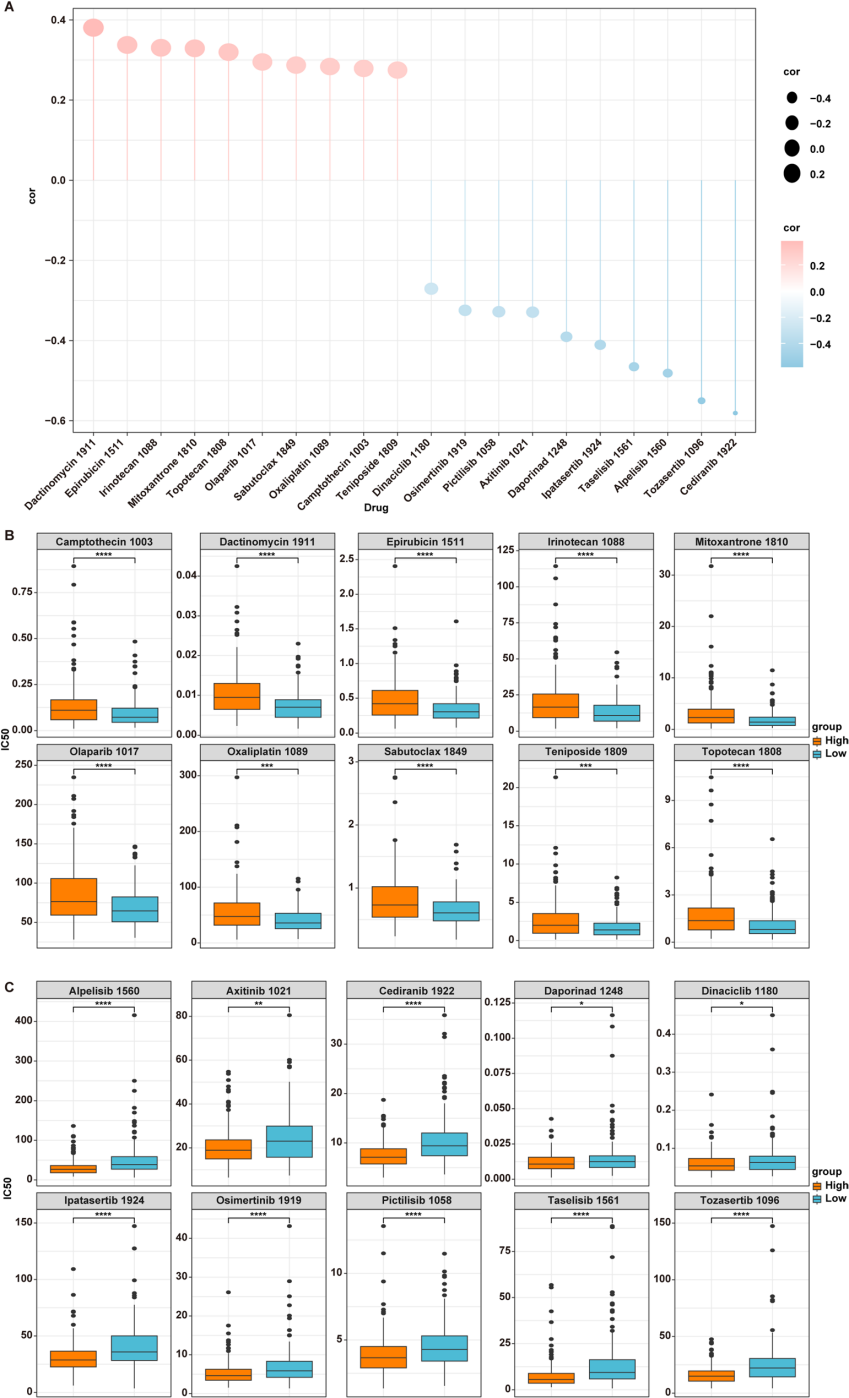
The correlation between PDIA4 and drug sensitivity. **A** The correlation between the IC50 values of 20 anti-tumor drugs and PDIA4 levels in the TCGA-OSCC cohort. Red: positive correlation; blue: negative correlation. **B**,** C** Differences in IC50 values of 20 drugs between the H-PDIA4 and L-PDIA4 groups. *P < 0.05; **P < 0.01; ***P < 0.001; ****P < 0.0001

Drug sensitivity was assessed by estimating the IC50 value via the “oncoPredict” package in R [31]. It has been shown that the negative correlation between gene expression and the drug’s IC50 suggests that patients with high gene expression are more sensitive to it [35]. Results revealed a notable negative correlation between PDIA4 expression and the IC50 values of 78 drugs, such as Cediranib, Tozasertib, Alpelisib, Taselisib, Ipatasertib, Daporinad, Axitinib, Pictilisib, Osimertinib and Dinaciclib (Fig. 6A-C, Table S4). These findings suggest that patients with high PDIA4 expression may be more sensitive to these agents.

Conversely, a significant positive correlation was observed between PDIA4 expression and the IC50 values of 53 anti-tumor drugs, such as Dactinomycin, Epirubicin, Irinotecan, Mitoxantrone, Topotecan, Olaparib, Sabutoclax, Oxaliplatin, Camptothecin and Teniposide (*P* < 0.05) (Fig. 6A-C, Table S4). This implies that patients exhibiting low PDIA4 expression may show increased sensitivity to these drugs.

### PDIA4 functions as an oncogene in OSCC

**Fig. 7:**
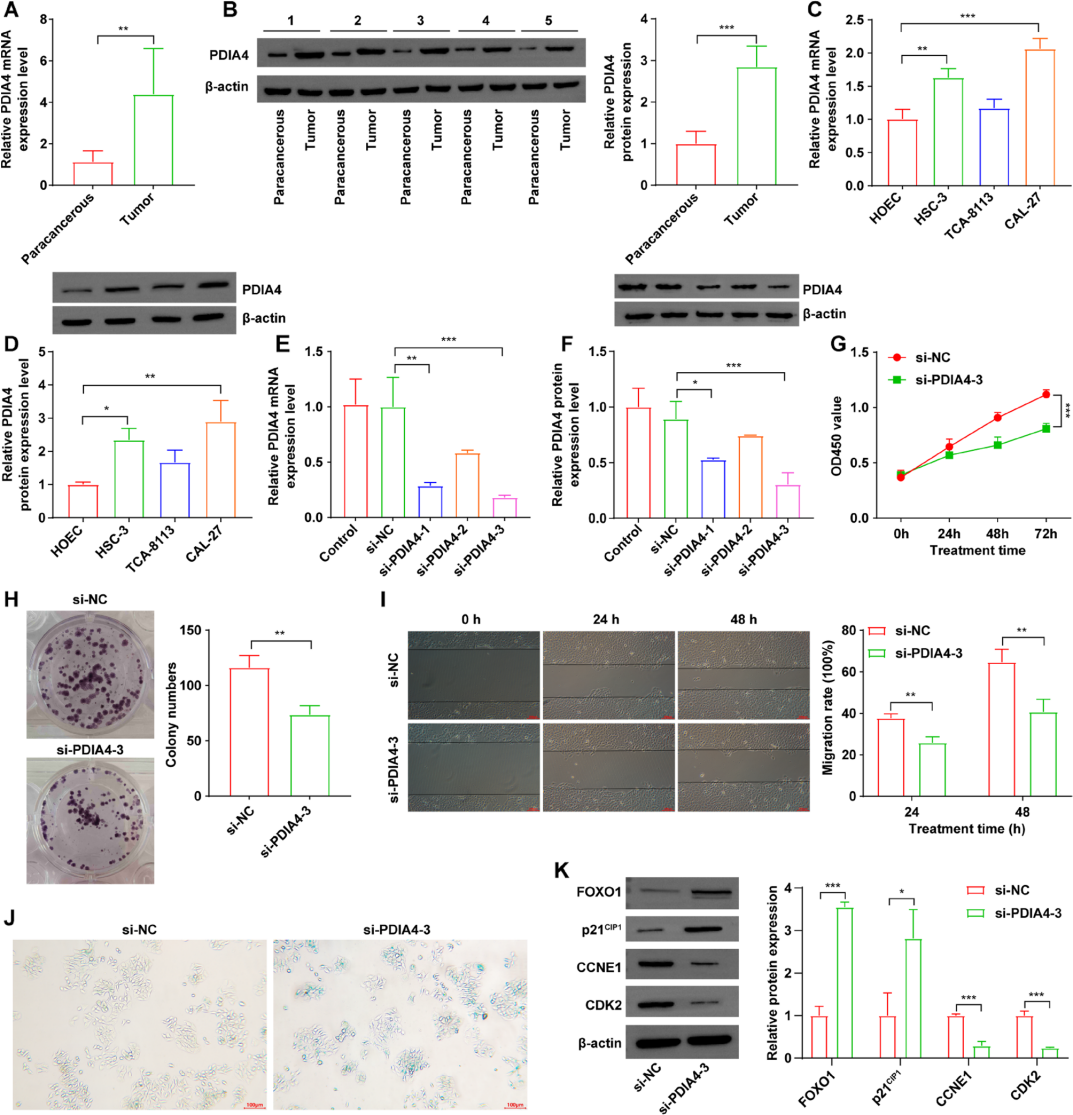
PDIA4 functions as an oncogene in OSCC. **A**,** B** RT-qPCR and western blot assays were conducted to determine PDIA4 levels in OSCC tissues. **C**,** D** RT-qPCR and western blot assays were performed to evaluate PDIA4 levels in OSCC cells. **E**,** F** CAL-27 cells were transfected with si-NC, si-PDIA4-1, si-PDIA4-2 and si-PDIA4-3, respectively. RT-qPCR and western blot assays were performed to assess PDIA4 levels in transfected cells. **G**,** H**,** I, J, K** CAL-27 cells were transfected with si-NC and si-PDIA4-3, respectively. Cell viability, proliferation, migration and cell senescence were evaluated by using **G** CCK-8, **H** colony formation, **I** wound healing and **J** SA-β-gal staining assays. **K** Western blot assay was applied to evaluate the expression levels of FOXO1, p21^CIP1^, CCNE1 and CDK2 in cells. *P < 0.05, **P < 0.01, ***P < 0.001

RT-qPCR and western blot analyses demonstrated a notable increase in PDIA4 levels within OSCC tissues and OSCC cells when compared to paracancerous samples and HOEC cells (Figs. 7 A-7D). As shown in Fig. 7 C and D, CAL-27 cells showed the highest PDIA4 expression among all three OSCC cells, which was selected for subsequent experiments.

Next, to explore the functional role of PDIA4 in OSCC cells, we employed specific siRNAs to decrease PDIA4 levels in CAL-27 cells, with si-PDIA4-3 showing the most pronounced reduction in PDIA4 expression when compared to si-NC group (Fig. 7E, 7 F). Furthermore, downregulation of PDIA4 resulted in a marked decrease in CAL-27 cell viability, proliferation and migration (Fig. 7G-I). Additionally, knockdown of PDIA4 prominently induced cellular senescence in CAL-27 cells (Fig. 7 J). Meanwhile, downregulation of PDIA4 considerably elevated the expression levels of FOXO1 and P21^CIP1^, and reduced the expression levels of CCNE1 and CDK2 in CAL-27 cells (Fig. 7 K). These results suggest that PDIA4 may exhibit a critical role as an oncogene in OSCC.

## Discussion

The potential of PDIA4 as a prognostic marker has been investigated across various types of cancers [36, 37]. Research indicates that elevated levels of PDIA4 correlated with unfavorable outcomes in several cancers, including glioma, lung cancer, and breast cancer [22, 36, 37]. In this study, we uncovered for the first time that PDIA4 expression obviously elevated in OSCC, and this elevation was recognized as an independent predictor of worse outcomes for OSCC patients. These findings imply that PDIA4 may serve as a potential prognostic indicator for OSCC patients.

In our investigation, we also assessed the functional role of PDIA4 in OSCC, and discovered that downregulation of PDIA4 notably hindered OSCC cell proliferation, migration, implying that PDIA4 may function as an oncogene in OSCC. To understand whether this oncogenic role is specific to OSCC or conserved across tumors, it is crucial to compare our findings with existing evidence. Interestingly, the reported functions of PDIA4 in cancer are highly context-dependent. For example, our results align with studies in glioblastoma [17] and cervical cancer [22], where PDIA4 also acts as an oncogene by promoting proliferation and is associated with poor survival. Conversely, in lung cancer, PDIA4 deficiency has been reported to enhance cell proliferation and migration [21], presenting a stark contrast to our findings. This divergence suggests that the functional role of PDIA4 is likely tissue-specific and influenced by a multitude of factors.

Mechanistically, it has been indicated by Wang et al. that PDIA4 facilitated the progression of glioblastoma through the PI3K/AKT/mTOR pathway [17]. Additionally, Kang et al. proposed that PDIA4 enhanced resistance to ferroptosis in renal cell carcinoma via the ATF4/SLC7A11 axis [38]. Nonetheless, the molecular mechanisms through which PDIA4 contributes to tumorigenesis remain unclear. Consequently, we conducted functional analysis to explore cellular signaling pathways in which PDIA4 might be implicated in OSCC. GSEA results showed that both FoxO and Hippo signaling pathways were obviously inactivated in H-PDIA4 group. These two pathways are crucial in tumor development [39, 40]. Available evidence indicates that the activation of the FoxO and Hippo signaling pathways can prevent tumor development [39, 40]. Therefore, we hypothesize that PDIA4 may promote OSCC development by impeding the modulation of the FoxO and Hippo signaling pathways, a mechanism that warrants further investigation. FOXO1 has been shown to exert tumor-suppressive effects by inducing cell cycle arrest and apoptosis [41]. In this study, we demonstrated that downregulation of PDIA4 notably increased the expression of FOXO1 and p21^CIP1^, but reduced the expression of CCNE1 and CDK2 in OSCC cells. Thus, we propose that the suppression of PDIA4 may inhibit OSCC progression, at least in part, by activating the FOXO1 pathway.

OSCC are highly immunogenic tumors that are frequently marked by a substantial presence of immune cell infiltration [42]. Various immune cell types play distinct roles in tumor progression [43]. For instance, certain immune cells, such as CD4 T cells, CD8 T cells and NK cells have the capacity to directly eliminate tumor cells; conversely, other immune cell types (e.g., Tregs) may facilitate tumor growth [44]. In addition, the elevated infiltration of CD8 T cells, CD4 T cells, and NK cells in the tumor microenvironment (TME) is linked to improved patient’s prognosis [45]. On the other hand, the presence of Tregs, which dampen the anti-cancer immune response, correlates with unfavorable prognosis [46]. Our investigation revealed that the infiltration of CD4 T cells, CD8 T cells, and NK cells had a negative correlation with the H-PDIA4 group, whereas Tregs exhibited a notable positive correlation with the H-PDIA4 group. These findings imply that the poor prognosis observed in patients in the H-PDIA4 group may relate to heightened infiltration of Tregs and diminished presence of anti-tumor immune cells. Additionally, we hypothesize that PDIA4 may influence the progression of OSCC by modulating the infiltration of immune cells, either directly or indirectly. This hypothesis warrants further investigation in subsequent studies.

Furthermore, certain immune cells (e.g. CD4 T cells) within the TME are involved in immune surveillance by recognizing and destroying tumor cells [47, 48]. However, the abnormal expression of ICs such as PD-1 on these immune cells can dampen their anti-tumor responses, enabling cancer cells to escape immune detection [49]. Currently, the TIDE score is utilized to assess the likelihood of tumor immune evasion, where a higher TIDE score indicates an increased probability of immune escape and diminished effectiveness of immunotherapy [50]. In this research, we observed that individuals in the H-PDIA4 group demonstrated elevated TIDE scores when compared to those in the L-PDIA4 group. These results imply that OSCC patients categorized in the H-PDIA4 group may exhibit lower responsiveness to immunotherapeutic interventions.

The drug sensitivity analysis revealed that patients with high PDIA4 expression showed increased sensitivity to Dinaciclib, a cyclin-dependent kinase inhibitor [51]. Dinaciclib has demonstrated antitumor activity across multiple cancer types [52, 53]. Consistent with this, Oner et al. reported that Dinaciclib inhibits OSCC progression by disrupting cell cycle progression [54]. In our study, downregulation of PDIA4 significantly reduced the expression of CDK2 and CCNE1 in OSCC cells, suggesting a functional role for PDIA4 in cell cycle regulation. Taken together, these findings imply that high PDIA4 expression may confer increased sensitivity to Dinaciclib in OSCC by sustaining the cell cycle progression (e.g., through regulating CDK2/CCNE1). Thus, PDIA4 may represent a promising predictive biomarker for Dinaciclib responsiveness in OSCC patients. However, the functional consequence of PDIA4’s correlation with Dinaciclib sensitivity remains speculative. Future validation using PDIA4-knockdown or PDIA4-overexpression models treated with Dinaciclib is crucial to definitively elucidate the underlying mechanism.

While this study provides valuable insights, there are still several limitations. First, although transcriptomic information from the TCGA database offers essential insights into patterns of gene expression, the analysis is susceptible to technical and biological biases [55]. Additionally, the limited number of normal samples compared to tumor samples in the TCGA cohort may also introduce statistical bias [56]. Therefore, further validation of PDIA4 expression patterns using larger, well-balanced clinical samples is warranted to ensure robust conclusions. Second, it has been shown that cancer cell plasticity drives cancer progression, metastasis and therapy resistance [57]. Although our study demonstrated that inhibiting PDIA4 suppresses the proliferation and migration of OSCC cells, the effect of PDIA4 inhibition on the plasticity of OSCC cells remains unclear and warrants further investigation in future research. Furthermore, the role of PDIA4 in OSCC are confirmed solely by in vitro data, necessitating further animal experiments for in vivo verification. Finally, E64FC26 is a potent pan-inhibitor of the PDI family [58]. In multiple myeloma mouse models, E64FC26 demonstrated synergy with bortezomib, prolonging survival without observable toxicity [59]. This underscores the significant roles of PDI family proteins in cancer. Future studies should explore the therapeutic potential of E64FC26 in OSCC or focus on developing novel small-molecule inhibitors targeting PDIA4 for clinical applications.

## Conclusion

The findings of our research confirmed that PDIA4 was elevated in both OSCC tissues and OSCC cells. Additionally, high levels of PDIA4 may be linked to poor prognosis of OSCC and reduced responsiveness to immunotherapy. Furthermore, downregulation of PDIA4 was capable of preventing OSCC cell proliferation and migration. This study may offer new insights into the role of PDIA4 in OSCC.

## Supplementary Information

Supplementary Material 1: Table S1 The results of GO and KEGG analyses on DEGs between H-PDIA4 and L-PDIA4 groups

Supplementary Material 2: Table S2 GSEA results between H-PDIA4 and L-PDIA4 groups.

Supplementary Material 3: Table S3 GSVA results between H-PDIA4 and L-PDIA4 groups.

Supplementary Material 4: Table S4 The correlation between PDIA4 and drug sensitivity.

Supplementary Material 5: Figure S1 Kaplan-Meier subgroup analysis. Kaplan-Meier survival analysis comparing the H-PDIA4 and L-PDIA4 groups in subgroups of the TCGA-OSCC cohort based on age (A, B), gender (C, D), and stage (E-H).

Supplementary Material 6.

## Data Availability

The original contributions presented in the study are acquired from The Cancer Genome Atlas (TCGA, https://tcga-data.nci.nih.gov/tcga/) database and Gene Expression Omnibus (GEO database, https://www.ncbi.nlm.nih.gov/geo/) database.
