# Supplementary figures and images for: Downregulation of PDIA4 inhibits proliferation and migration in human oral squamous cell carcinoma
Source: Hereditas. 2025 Nov 3;162:222. doi: 10.1186/s41065-025-00594-2 (PMC12581318; doi:10.1186/s41065-025-00594-2)

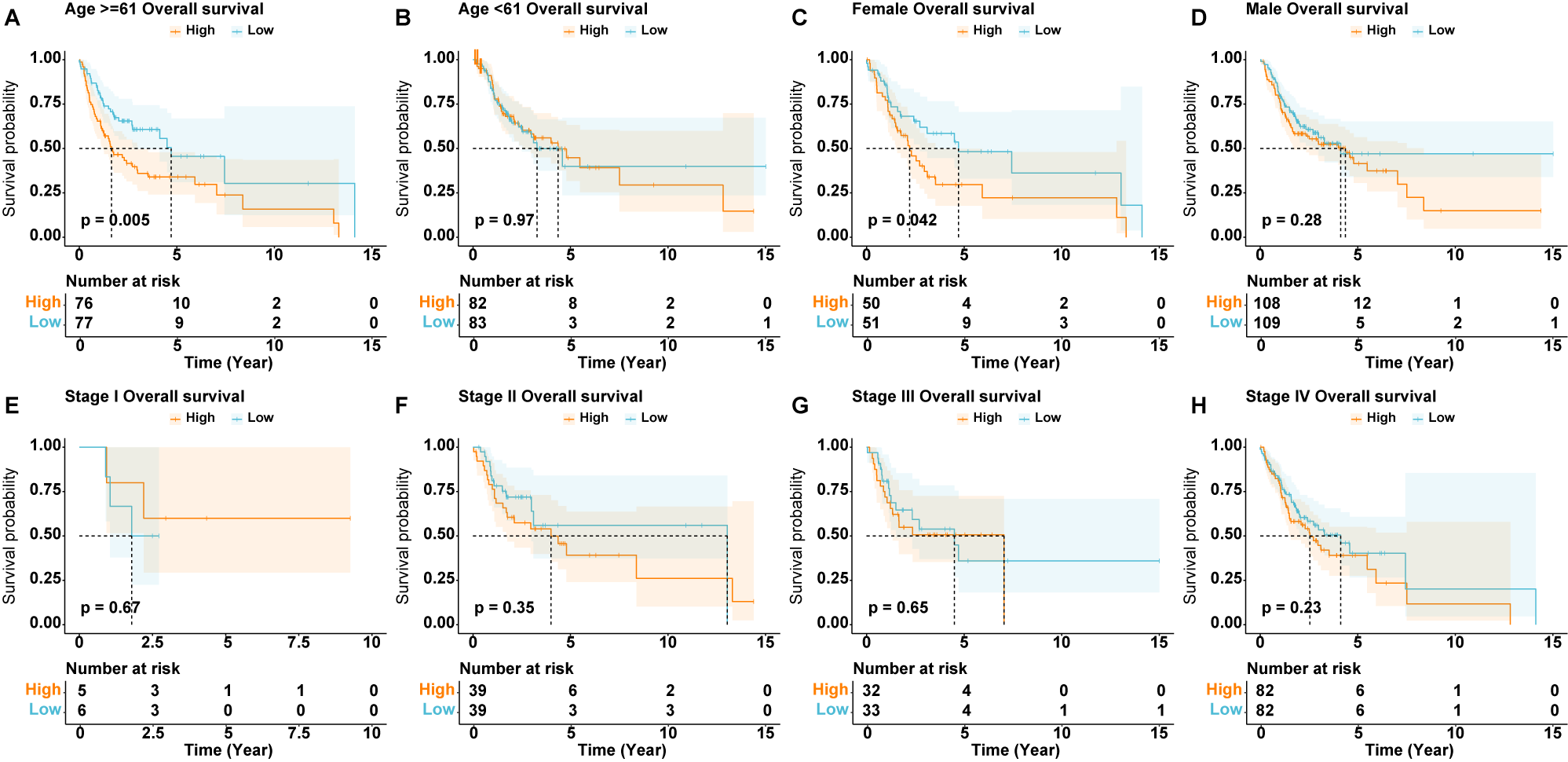

Supplement: Supplementary file 5 — Supplementary Material 5: Figure S1 Kaplan-Meier subgroup analysis. Kaplan-Meier survival analysis comparing the H-PDIA4 and L-PDIA4 groups in subgroups of the TCGA-OSCC cohort based on age (A, B), gender (C, D), and stage (E-H). [file 41065_2025_594_MOESM5_ESM.tif]
