# Supplementary material for: Downregulation of PDIA4 inhibits proliferation and migration in human oral squamous cell carcinoma
Source: Hereditas. 2025 Nov 3;162:222. doi: 10.1186/s41065-025-00594-2 (PMC12581318; doi:10.1186/s41065-025-00594-2)

Figure 7B

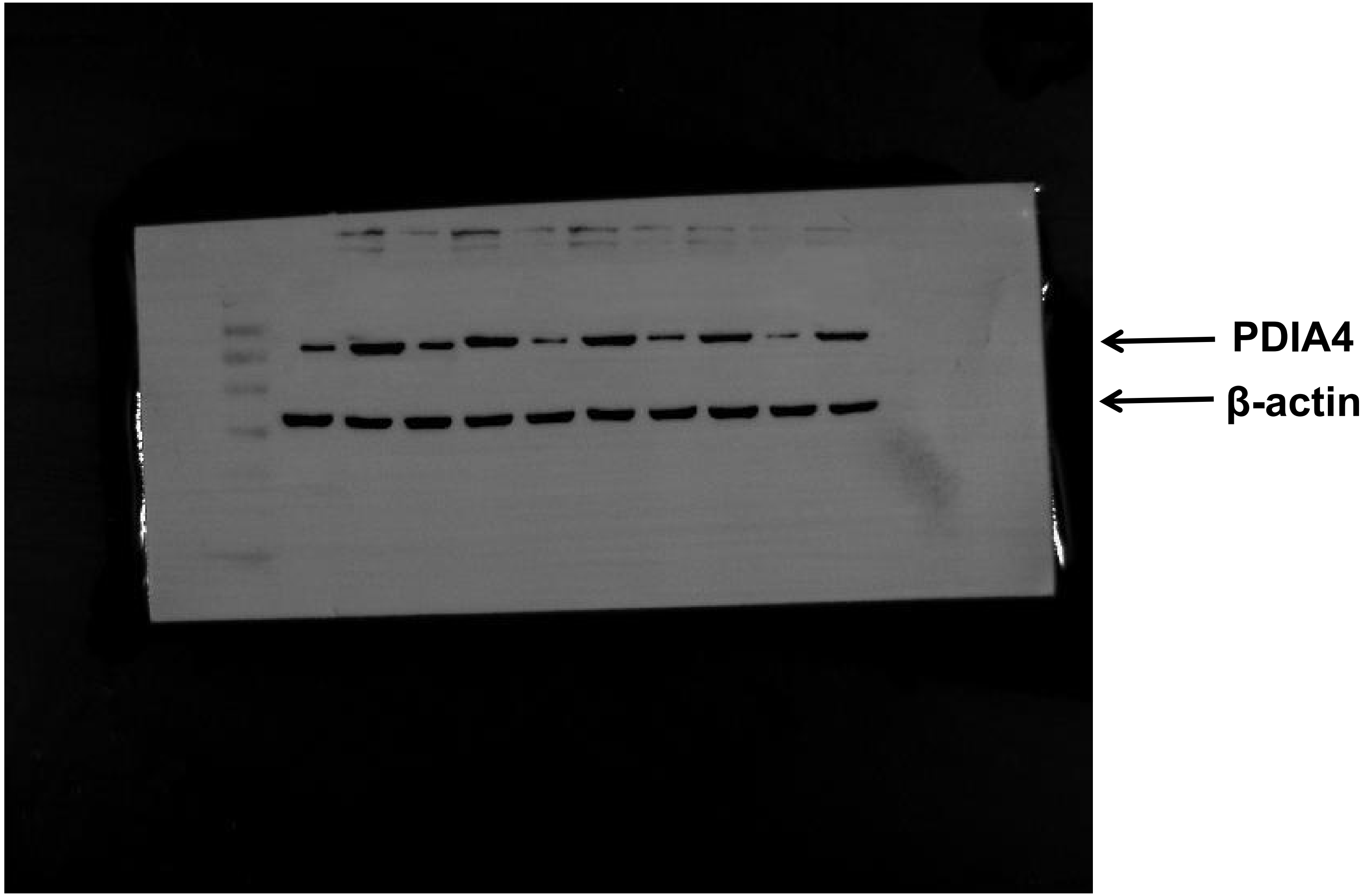

Figure 7D

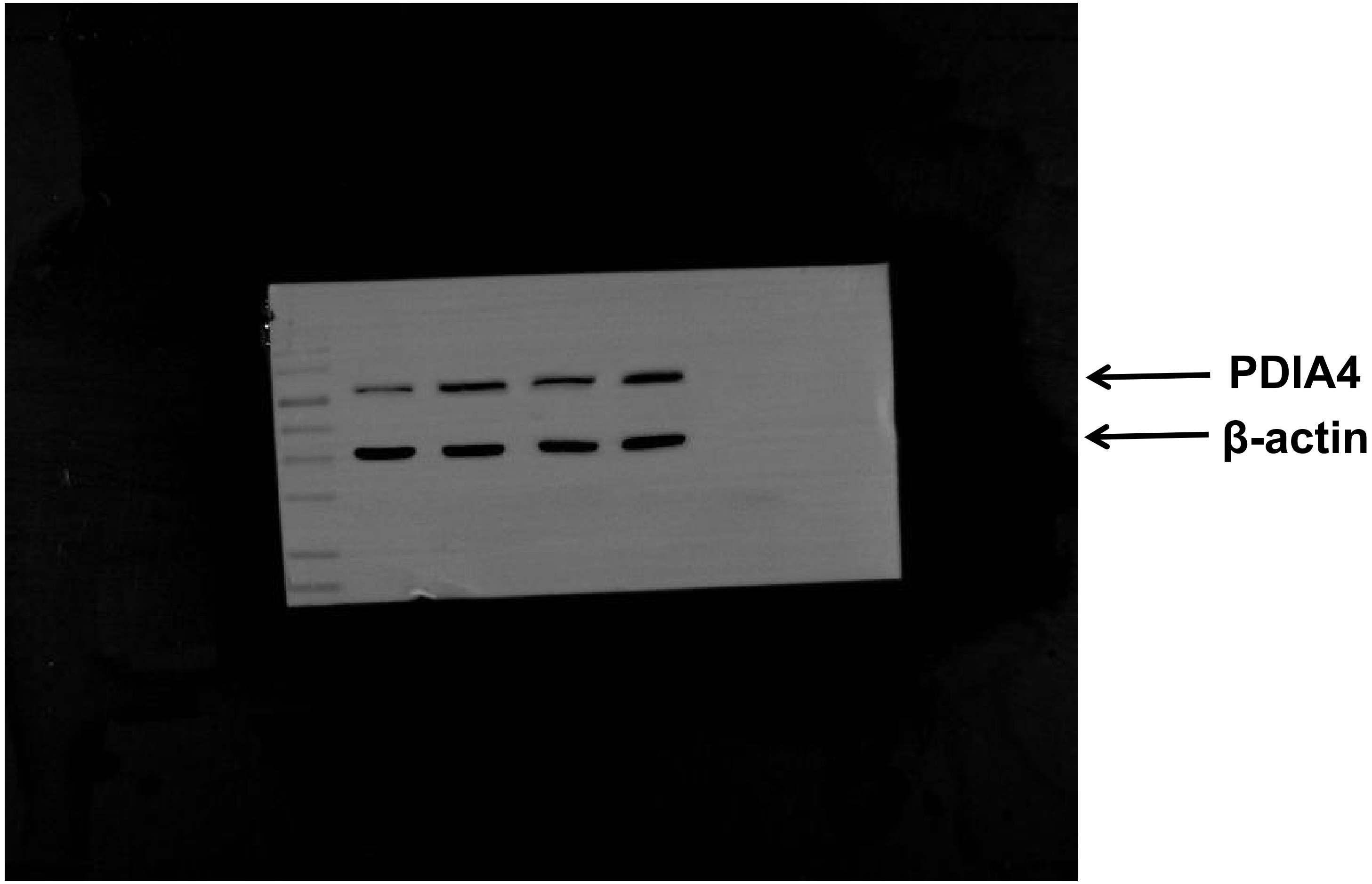

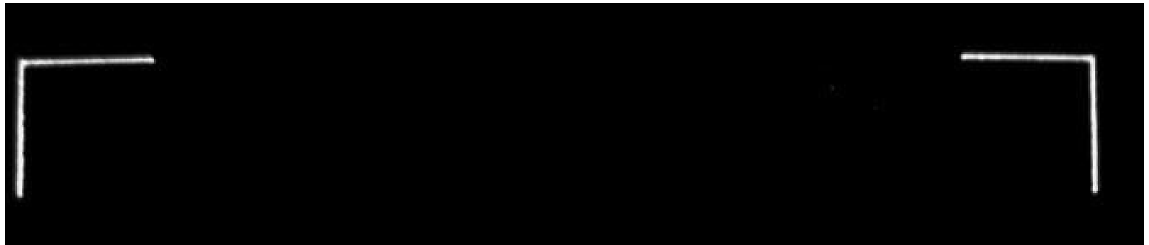

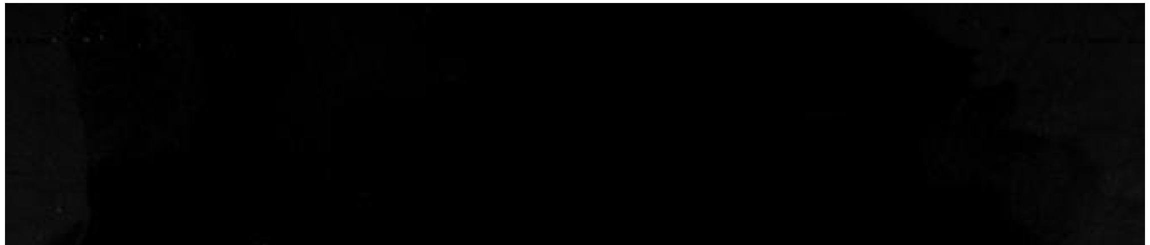

Figure 7F

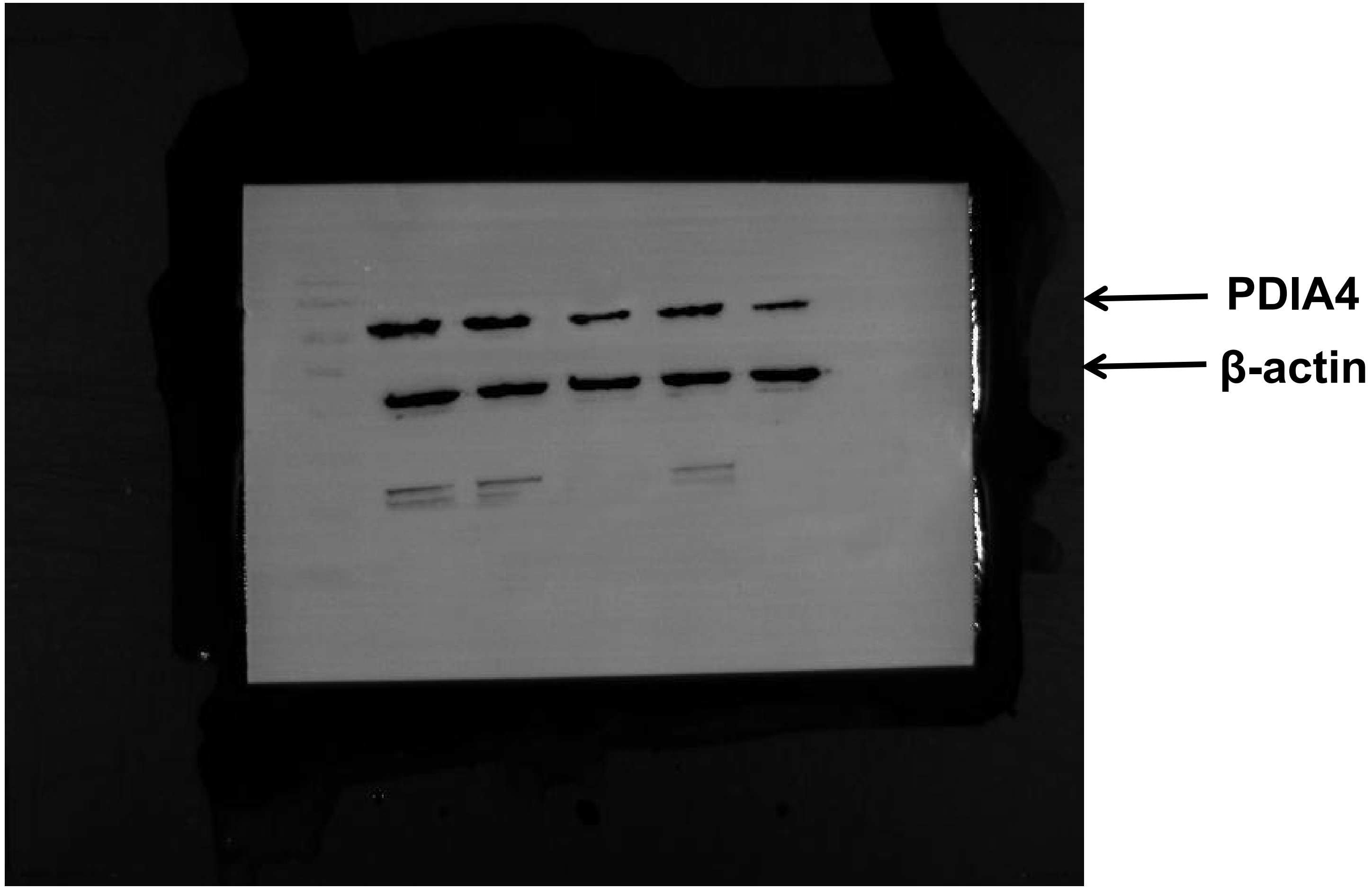

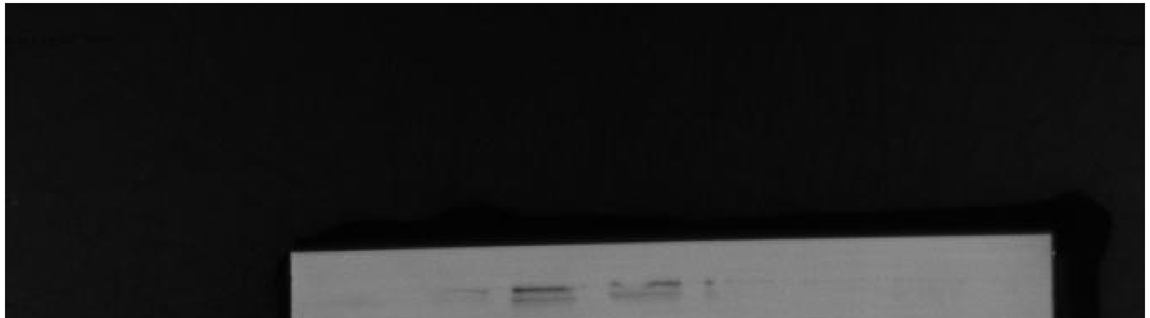

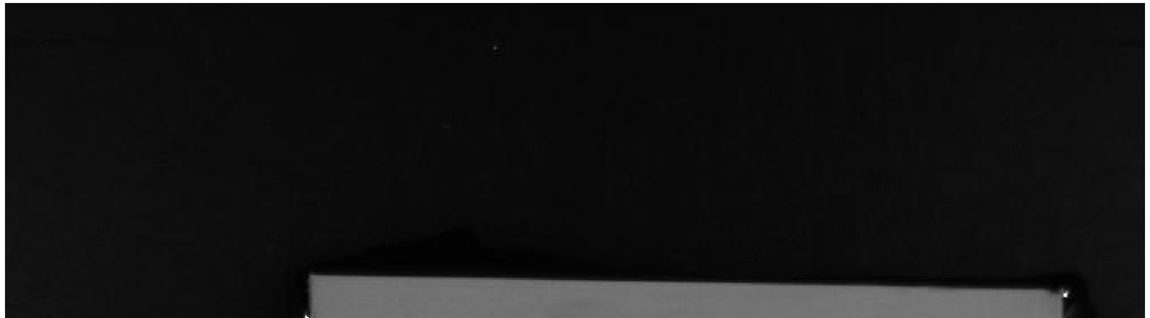

Figure 7K

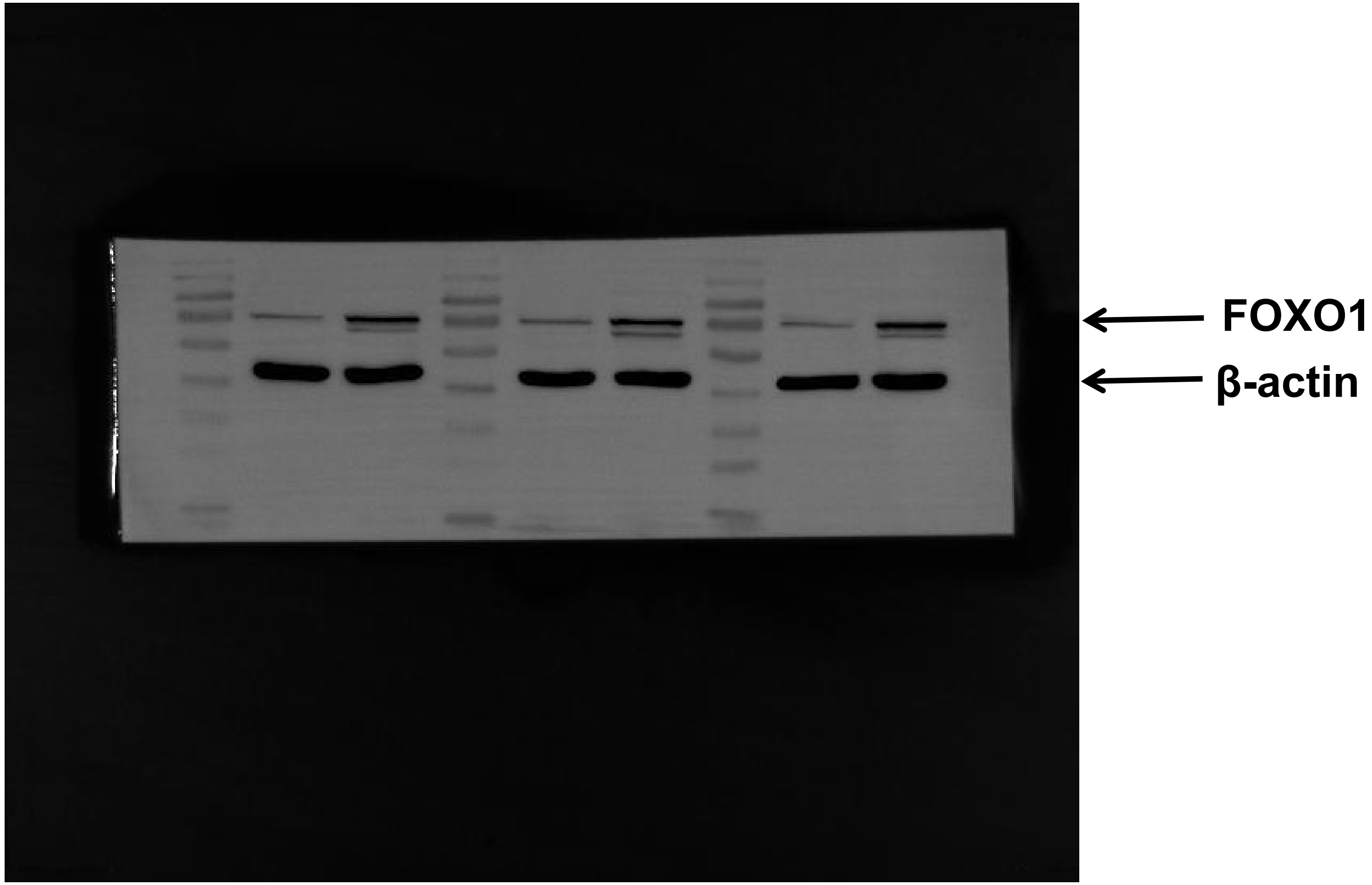

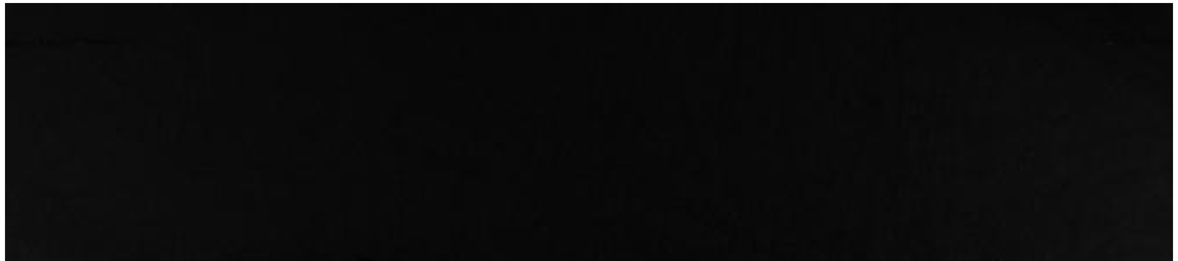

Figure 7K

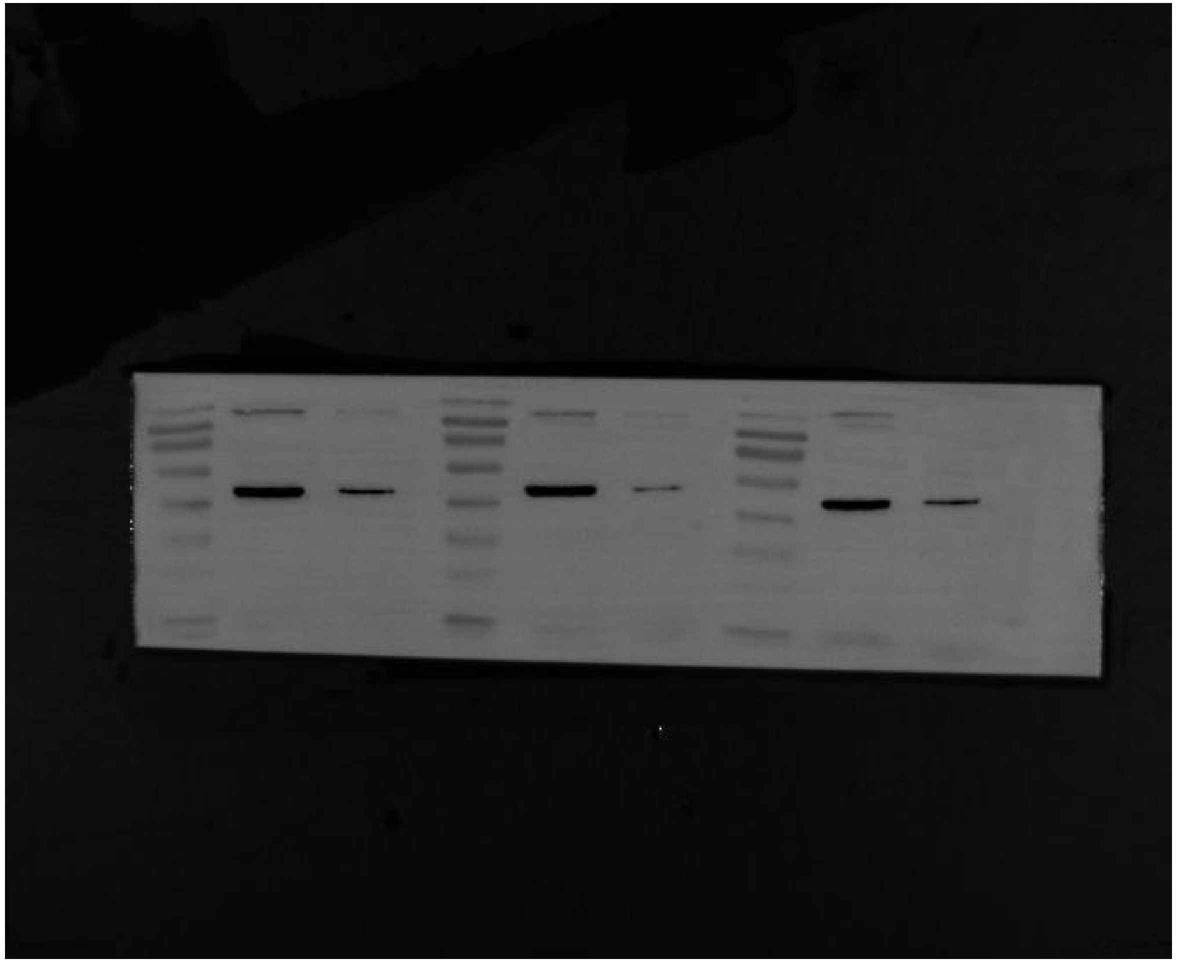

CCNE1

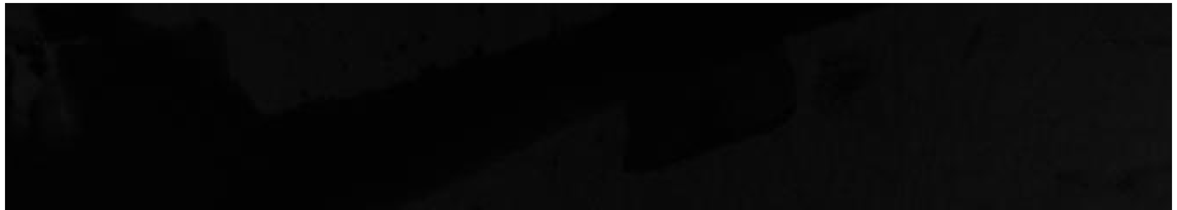

Figure 7K

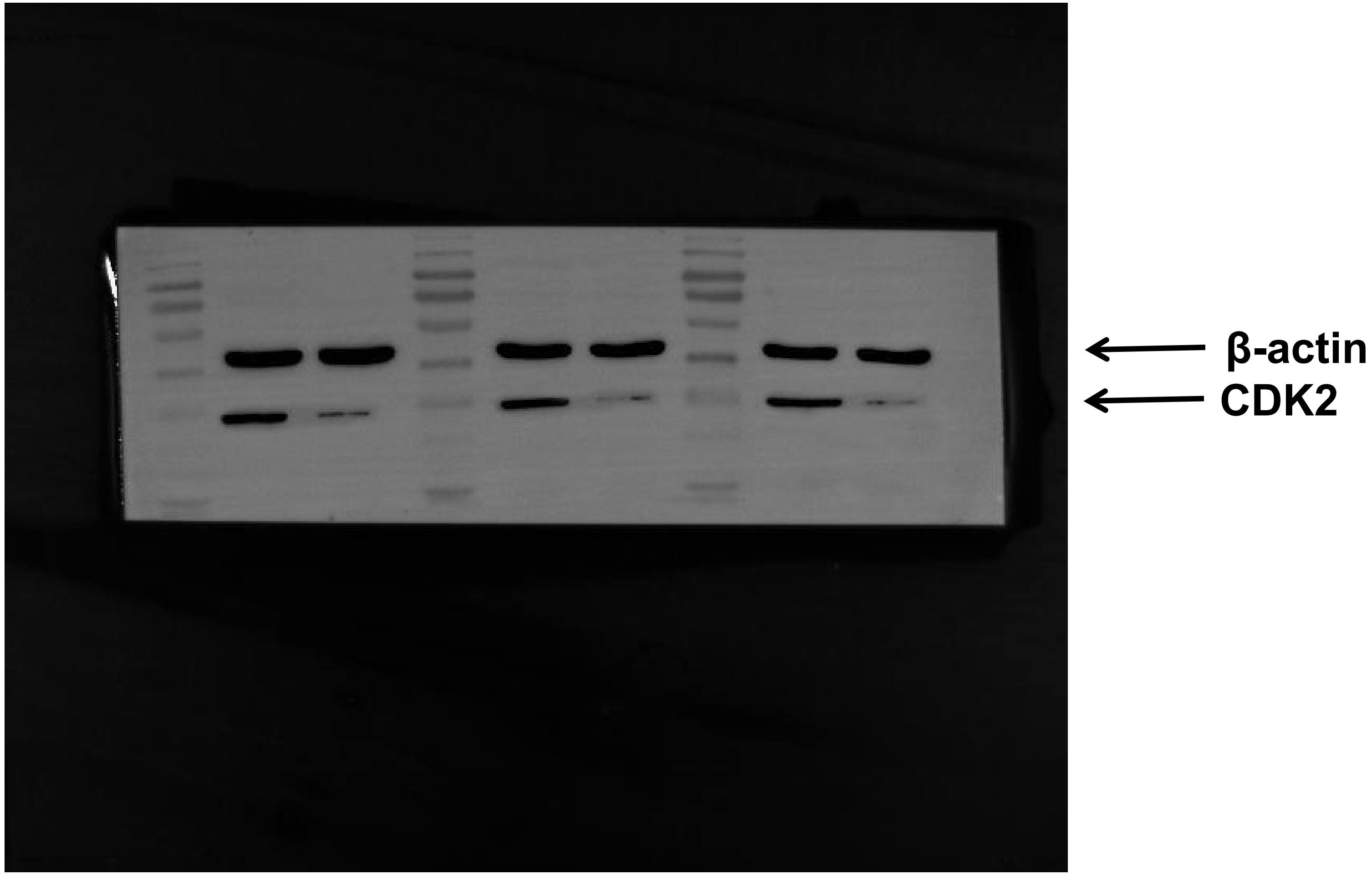

Supplement: Supplementary file 6 — Supplementary Material 6. [file 41065_2025_594_MOESM6_ESM.pdf]
